# Valorization and Potential Antimicrobial Use of Olive Mill Wastewater (OMW) from Italian Olive Oil Production

**DOI:** 10.3390/antiox11050903

**Published:** 2022-05-04

**Authors:** Eleonora Russo, Andrea Spallarossa, Antonio Comite, Marcello Pagliero, Patrizia Guida, Vittorio Belotti, Debora Caviglia, Anna Maria Schito

**Affiliations:** 1 Department of Pharmacy, University of Genova, Viale Benedetto XV, 3, 16132 Genoa, Italy; spallarossa@difar.unige.it; 2 Department of Chemistry and Industrial Chemistry, University of Genova, Via Dodecaneso, 31, 16146 Genoa, Italy; antonio.comite@unige.it (A.C.); marcello.pagliero@unige.it (M.P.); 3 Department of Phisics, University of Genova, Via Dodecaneso, 31, 16146 Genoa, Italy; patriziaguida@yahoo.it; 4 Department of Mechanical, Energy, Management and Transport Engineering, University of Genova, Via alla Opera Pia, 15, 16100 Genoa, Italy; vittorio.belotti@unige.it; 5 Department of Integrated Surgical and Diagnostic Sciences, University of Genova, Viale Benedetto XV, 6, 16132 Genoa, Italy; debora.caviglia@edu.unige.it (D.C.); amschito@unige.it (A.M.S.)

**Keywords:** olive mill wastewater, antioxidant activity, polyphenols, membrane processes, antibacterial activity, circular economy

## Abstract

The production of olive oil generates olive mill wastewater (OMW) which essentially derives from the processing, treatment and pressing of olives in mills. Traditional milling processes require a quantity of water varying between 40 and 120 L per quintal of pressed olives, generating a considerable amount of wastewater. It is thus necessary to reduce process water and enhance its use to implement the concept of a circular economy. To this end, our preliminary work was dedicated to water purification by means of suitable and efficient filtration systems. The microfiltered OMW was firstly concentrated through reverse osmosis. Then, an additional concentration step was carried out via vacuum membrane distillation using hydrophobic hollow fiber membranes. The application of the membrane-based processes allowed the recovery of a purified water and the concentration of valuable polyphenols in a smaller volume. The different fractions obtained from the purification have been tested for the determination of the antioxidant power (DPPH assay) and dosage of polyphenols (Folin–Ciocalteu assay) and were characterized using IR spectroscopy. All samples showed relevant antioxidant activity (percentage range: 10–80%) and total phenolic content in the 1.5–15 g GAE/L range. The obtained fractions were tested for their antimicrobial effect on numerous clinical isolates of Gram-positive and Gram-negative species, resistant and multi-resistant to current antibiotic drugs. OMW samples showed widespread activity against the considered (phyto)pathogens (MIC range 8–16 mg/mL) thus supporting the value of this waste material in the (phyto)pharmaceutical field.

## 1. Introduction

**Figure 1:**
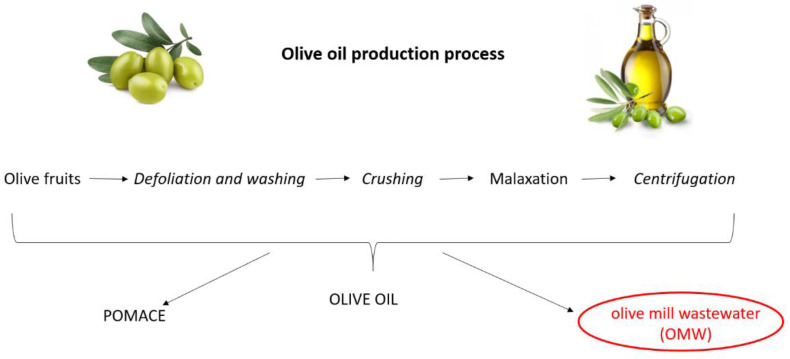
Olive oil production process.

Olive oil is the primary source of fat in the Mediterranean diet, the nutritional benefits of which are recognized globally [1]. Triglycerides represent the major components of olive oil (98–99%) [2]. Moreover, it contains more than 200 minor components including sterols, waxes, tocopherols, carotenes and chlorophylls, phenolic and volatile compounds [3]. Olive oil production is a multistep procedure that includes defoliation and washing of the olive fruits; crushing of the olives by mills, hammers or blades and, after malaxation, a final solid–liquid extraction (Figure 1). The modern method of olive oil extraction involves the use of a horizontal centrifuge (the so-called decanter) that separates the materials (namely pomace, olive oil and vegetation water) on the basis of their different density. Pomace is a solid part deriving from the pulp of the olives and represents a source of income for millers as it can be used for the production of pomace oil or fuel. Olive mill wastewater (OMW) essentially consists of washing and process waters, as well as the aqueous fraction of drupe juices. Depending on the extraction system used (i.e., three-, two- or two-and-half-phase decanter), the volumes of OMW can vary from 40 to 120 L per quintal of pressed olives, generating a considerable amount of wastewater [4]. A conventional milling procedure yields 20% olive oil, 30% solid residues and about 50% OMW [5].

OMW has a dark-brown color that can turn black and is characterized by a typical, rather intense smell, reminiscent of that of olives. It consists of an aqueous phase in which organic substances (e.g., reducing sugars, organic acids, polyalcohol) and inorganic elements (e.g., potassium, phosphorus, calcium) are dissolved and a suspended part containing the solid vegetable material not filtered during the oil separation phase. The phenolic content of OMW includes (hydroxy)tyrosol, phenolic acids (e.g., caffeic acid), secoiridoids (e.g., oleuropein, p-DHPA-EA), flavonoids (e.g., apigenin, quercetin) and lignans; see [6] and references therein. OMW composition depends on different factors including the extraction system, olive cultivar and biometric values of the olive fruit [7,8,9].

OMW has significant environmental impact and represents one of the major industrial effluents [10]. OMW bears a high organic load with a high quantity of (phyto)toxic compounds (e.g., phenols). Moreover, chemical oxygen demand (COD) [11] and biochemical oxygen demand (BOD) values of OMW can be as high as 220 and 100 g/L, respectively [12]. The high levels of COD and BOD reflect the concentration of organic pollutants in wastewater and are associated with the environmental impact of OMW. The direct discharge of OMW into soil or rivers could cause damage to the flora or depletion of clean water reservoirs [13]. From a legislative point of view, liquid wastes from olive oil production fall under the Urban Waste Water Treatment Directive [14], which also regulates treatment and discharge of wastewater from the olive oil industry sector [15]. However, a specific EU legislation on OMW management does not exist, and each EU production country has implemented its own national guidelines [16]. According to the Italian legislative regulations [17,18,19], OMW must be disposed of by spreading on the agricultural ground or by agronomic use only after the reduction in the COD and BOD content (emission limits for urban wastewater plants: COD 125 mg/L, BOD 25 mg/L). 

To reduce its environmental impact, a number of physicochemical, biological and combined processes have been investigated for the treatment of OMW. One of the possible ways to treat OMW and concentrate its phenolic compounds is membrane filtration. Most of the investigations on the applications of membrane processes to the treatment of olive oil vegetation waters focused on pressure-driven membrane processes (e.g., microfiltration, ultrafiltration, reverse osmosis and nanofiltration), but in recent years emerging membrane processes such as membrane distillation are under investigation [20,21]. Recently, some of us reported an innovative pressure-driven membrane process for the treatment of OMW that consists firstly of microfiltration (MF) followed by a reverse osmosis (RO) stage [22]. To further concentrate the RO fraction (and the polyphenol content therein), vacuum membrane distillation (VMD) using hydrophobic hollow fiber membranes was carried out. Notably, in recent decades, particular attention has been devoted to the possibility of fractionating or recovering polyphenolic compounds as a strategy for the valorization of byproducts from a circular economy perspective [23,24,25,26]. Polyphenolic compounds recovered from waste products showed pharmaceutically relevant properties such as antioxidant, anti-allergic, anti-inflammatory, anti-tumor and anti-hypertensive effects. Moreover, these compounds have an antimicrobial activity that could be exploited both in agriculture for the fight against phytopathogens and in the identification of new therapeutic agents active against human antibiotic-resistant pathogens [27,28]. In this regard, *Enterococcus faecium, Staphylococcus aureus, Klebsiella pneumoniae, Acinetobacter baumannii, Pseudomonas aeruginosa* and *Enterobacter cloacae* (ESKAPE) bacteria are a group of antibiotic-resistant pathogens that globally represent the major cause of life-threatening nosocomial infections.

**Figure 2:**
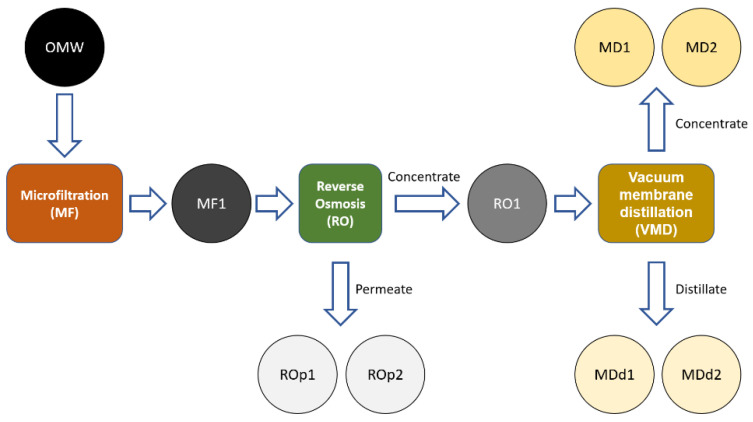
Schematic representation of OMW purification process. Analyzed samples: MF1: sample collected after the MF. ROp1: permeate sample collected in the middle of RO purification. ROp2: final permeate sample of RO purification. MD1: initial concentrate sample of VMD. MD2: final concentrate sample of VMD. MDd1: initial distillate sample of VMD. MDd2: final distillate sample of VMD.

Herein, we report the physicochemical characterization (conductivity and infrared spectroscopy), the total polyphenols (Folin–Ciocalteu test) and the antioxidant activity (DPPH assay) of different MF, RO and VMD fractions derived from OMW (Figure 2).

Additionally, the antibacterial properties of OMW fractions were studied against 39 bacterial strains, including multi-resistant pathogens belonging to the “ESKAPE” group. The acquired results provide the basis for a re-evaluation of OMW that, within the concept of a circular economy, should not be considered as waste material of the olive pressing process but instead an important source of (phyto)pharmaceutic compounds. The modernization and restructuring of the oil mills with the installation of appropriate OMW filtering units would increase the economic performance of factories in both reducing water consumption and producing valuable material with antioxidant and/or antibacterial potential.

## 2. Materials and Methods

### 2.1. Chemicals

All solvents, DPPH (2,2-diphenyl-1-picrylhydrazyl), Trolox (6-hydroxy-2,5,7,8-tetramethylchroman-2-carboxylic acid), Folin–Ciocalteu phenol reagent and gallic acid used as a reference standard were purchased from Sigma-Aldrich (Milan, Italy). The Milli-Q-system (Millipore SA, Molsheim, France) was used to produce deionized water.

### 2.2. Plant Material and OMW Used

The olives belong to the Taggiasca cultivar. The trees were grown in the province of Imperia (Liguria, North Italy), aged > 100 years, with an approximated olive production of 20 kg each. The soil is calcareous and alkaline with fine sand on the surface and friable rock in the subsoil. The plants were not irrigated and received common organic-mineral fertilizer every year (35 kg/tree; N, P and K in the ratio 20:10:10). The temperature range in the area was 0–35 °C. According to Regione Liguria data, mean rain values in the province of Imperia were 783 and 792 mm in 2018 and 2019, respectively. The olives were harvested in 2019 (autumn olive campaign) 180 days after blossom. Olives were characterized by the following biometric values: mean weight, 3 g; pulp/stone ratio, 25%; oil content, 27%; water content, 50%. Sample MF2 was derived from the 2018 olive campaign. The olives were processed (300 tons/year) by a semi-automatic three-phase olive processing plant located in Dolcedo (Imperia, Italy). In the malaxation phase water was added (10–20% by weight of olive paste). A total of 0.5 m^3^ of fresh olive mill wastewater was collected on site from immediately processed olives and stored at 4 °C before analysis [29]. The analyses were carried out a week after the olive processing.

### 2.3. Filtration Procedure

An overall volume concentration factor of 14.5 was achieved by combining different membrane processes. The volume concentration factor is defined as the ratio between the initial volume of the feed and the final volume of the concentrate after the filtration. Microfiltration (MF) was used as a pretreatment step before reverse osmosis to clarify the wastewater and to remove the suspended solids. Reverse osmosis (RO) and subsequently membrane distillation (MD) were applied to concentrate the OMW. The detailed procedure of filtration and concentration by ultrafiltration (UF) and RO is reported in a previous work [22]. The OMW was previously filtered onto a non-woven filter (about 200 µm) to remove the coarser particulate matter. The OMW was then microfiltered in a pilot plant using ceramic membranes (M-3P1940 module with 3 Membralox EP19-40 membranes, Pall Corp., Port Washington, NY, USA). The microfiltered OMW was concentrated through reverse osmosis of a factor of about 7.3 (380 L of permeate over 440 L of feed). The reverse osmosis membrane used was the SEA1-4040 supplied by Oltremare S.p.A (Fano, Italy), and the filtration conditions were 24 °C +/− 4 °C, 40 bar in concentration mode. As a further concentration step, a vacuum membrane distillation (VMD) was applied. The details of the laboratory plant for testing are reported in previously published papers [30,31]. A total of 15 hollow fiber membranes made of polypropylene (Accurel PP S6/2, Membrana, Germany) with a length of 30 cm were arranged in a module where the fiber extremities were potted with an epoxy resin in a PVC tube. The main membrane characteristics have been reported in another work [32]. The liquid flowed on the outer side of the hollow fiber membranes, and a vacuum (30 mbar) was applied from the membrane lumen. The contact area of the membrane module was 0.0382 m^2^. The distillation was performed at about 30 °C until a feed volume reduction of a factor 2 was obtained. The following samples were obtained: MF1 (sample collected after the MF); ROp1 (permeate sample collected in the middle of RO purification); ROp2 (final permeate sample of RO purification); MD1 (initial concentrate sample of VMD); MD2 (final concentrate sample of VMD); MDd1 (initial distillate sample of VMD); MDd2 (final distillate sample of VMD).

### 2.4. Determination of Electric Conductivity and Elemental Composition of Isolated Fractions

Electrical conductivity was measured through a Hanna EC215^TM^ conductivity meter equipped with a Hanna HI76303^TM^ probe. 

The liquid samples poured in a ceramic crucible were dried at 105 °C in a ventilated oven and successively at 600 °C in a furnace. The inorganic residue after calcination was collected and immobilized on a microscope stub to be sputtered with carbon to impart the necessary electrical conductivity for the electron microscopy analysis. A field emission scanning electron microscope (Zeiss Supra 40VP, Carl Zeiss, Germany) equipped with an energy dispersive X-ray microanalysis detector (EDX) was used to analyze some samples areas.

### 2.5. Fourier Transform Infrared Spectroscopy

The infrared spectra were acquired on an FTIR-ATR Jasco spectrophotometer (JASCO 4700, JASCO Corporation) at 4 cm^−1^ resolution, 50 scans, in the 4000–400 cm^−1^ spectral range using the liquid thin layer method at a temperature of 20 °C (in the dark). Each sample had 15 spectra recorded, and each measurement was repeated three times from each OMW sample.

### 2.6. DPPH Radical-Scavenging Activity

The antioxidant activity of the OMW was measured using the DPPH antioxidant assay. The DPPH assay is based on the bleaching rate of the stable radical 2,2-diphenyl-1-picrylhydrazyl (DPPH) [33]. A total of 0.1 mL of OMW was mixed with 3.9 mL of DPPH methanol solution (65 µM). Absorbance was measured at 517 nm after reacting for 30 min in the dark. The linear calibration curve was obtained using Trolox standards (ranging between 20 and 200 mg/L, R^2^ = 0.9955). The result was calculated as Trolox equivalent in mg/L, and the percentage of antioxidant activity (AA%) was calculated from the ratio of decreasing absorbance of sample solution (A_0_ – A_s_) to absorbance of blank DPPH solution (A_0_), as expressed in Equation (1) [34].

(1) $$\mathrm{AA}\%=\frac{A0-\mathrm{As}}{A0}\cdot 100$$

All analyses were performed in triplicate (*n* = 3), and values are given ± standard deviation (SD).

### 2.7. Folin–Ciocalteu Spectrophotometric Determination

The total polyphenol (TP) contents in OMW were determined using the Folin–Ciocalteu (FC) spectrophotometric method [35]. Absorbance was measured at 750 nm. TPs were quantified from a calibration curve prepared with gallic acid standard solutions in concentrations ranging from 20 to 80 mg/L (R^2^ = 0.9988) and expressed as g of gallic acid equivalent for OMW liter (g GAE/L) [36]. All analyses were performed in triplicate (*n* = 3), and values are given ± standard deviation (SD).

### 2.8. Antibacterial Activity of OMW

#### 2.8.1. Bacterial Strains

A total of 39 isolates, belonging to the Gram-positive and Gram-negative species, were used in this study. All with the exception of the strain of *Pseudomonas syringae pv*. tomato, kindly donated by Dr. Giovanni Minuto of the Centro di Sperimentazione e Assistenza Agricola (CERSAA) of Albenga (SV), Italy, were clinical strains isolates, belonging to a collection obtained from the School of Medicine and Pharmacy of University of Genoa (Italy), and identified by VITEK^®^ 2 (Biomerieux, Firenze, Italy) or matrix-assisted laser desorption/ionization time-of-flight (MALDI-TOF) mass spectrometric technique (Biomerieux, Firenze, Italy). Of the tested 16 Gram-positive organisms, eight strains belonged to the *Enterococcus genus*, (four *Enterococcus faecalis*, three of which were resistant to vancomycin (VRE); four *E. faecium*, two of which were VRE; eight strains pertained to the *Staphylococcus genus*, including four methicillin-resistant *S. aureus* (MRSA) and four methicillin-resistant *S. epidermidis* (MRSE) all of which were also resistant to linezolid). Regarding the 23 Gram-negative isolates, 9 strains were *Enterobacteriaceae*: 3 *Escherichia coli* (one was a fully susceptible strain to all antibiotics tested, 1 was a *Klebsiella pneumoniae* Carbapenemase (KPC)-producing strain, 1 was a New Delhi metallo-beta-lactamase (NDM) producer), 1 *Morganella morganii*, 1 *Providencia stuartii*, 1 *Serratia marcescens* and 3 KPC-producing *Klebsiella pneumoniae* isolates. Fourteen strains belonged to the non-fermenting group: nine *Pseudomonas aeruginosa* strains including strain 265 (MDR and resistant also to colistin) and strains 1, 2v, 19v, 16b, 12b and 8g (isolated from cystic fibrosis patients and MDR), one *P. syringae*, two *Stenotrophomonas maltophylia* (all resistant to sulfamethoxazole-trimethoprim) and two MDR *Acinetobacter baumannii*. According to the literature [37], our definition of MDR organism includes those isolates with diminished susceptibility to at least one antimicrobial drug in three or more antimicrobial categories.

#### 2.8.2. Determination of Minimum Inhibitory Concentration (MIC) and Minimum Bactericidal Concentration (MBC) of OMW

To investigate the antimicrobial activity of OMW samples, their minimum inhibitory concentrations (MICs) were determined by following the microdilution procedures detailed by the European Committee on Antimicrobial Susceptibility Testing EUCAST [38]. Briefly, after overnight incubation, cultures of bacteria were diluted to yield a standardized inoculum of 1.5 × 10^8^ CFU/mL. Appropriate aliquots of each suspension were added to 96-well microplates containing the same volumes of serial 2-fold dilutions (ranging from 125 to 2 mg/mL) of each OMW sample to yield a final concentration of about 5 × 10^5^ cells/mL. After 24 h of incubation at 37 °C, the lowest concentration of sample that prevented visible growth was recorded as the MIC. All MICs were obtained in triplicate, the degree of concordance in all the experiments was 3/3 and the standard deviation (±SD) was less than 10%. 

The minimum bactericidal concentration (MBC), defined as the lowest concentration of a drug that results in killing 99.9% of the bacteria being tested, was determined for each OMW sample by subculturing the broths used for MIC determination. A total of 10 μL of the culture broths of the wells corresponding to the MIC and to higher MIC concentrations was plated onto fresh MH agar plates and further incubated at 37 °C overnight. The highest dilution that yielded no bacterial growth on the agar plates was taken as the MBC. All tests were performed in triplicate, and the results were expressed as the mode.

### 2.9. Statistical Analysis

Each sample was analyzed in triplicate, and Folin–Ciocalteu and DPPH data were subjected to analysis of variance (ANOVA) using JMP^®^ software Trial 16.2.0 for Windows 10 (JMP Italy, Via Darwin 20/22 20143 Milano). Wherever F values were significant, Tukey’s test was used for means comparison. Significance was defined at *p* < 0.001.

## 3. Results and Discussion

### 3.1. OMW Concentration from Membrane Processes

**Figure 3:**
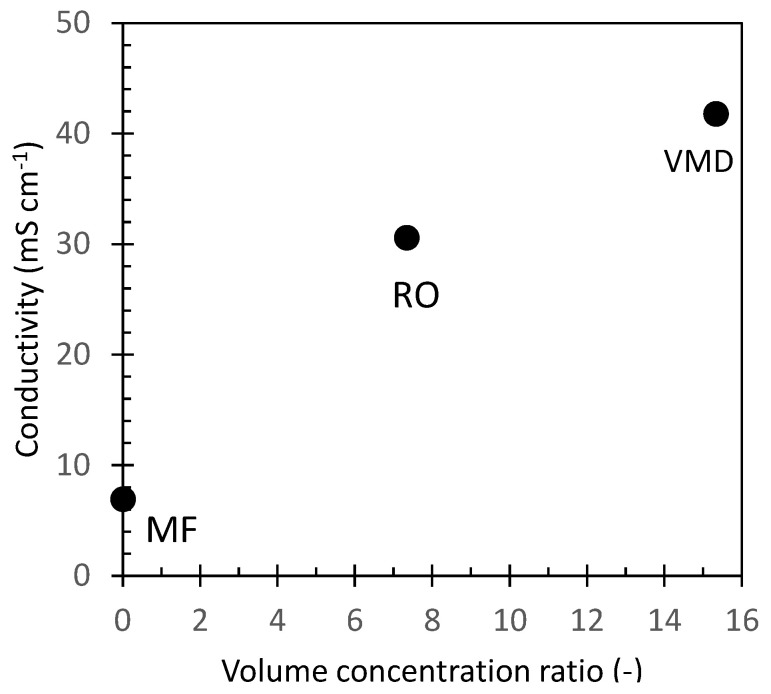
OMW conductivity against the volume concentration ratio achieved at each treatment step.

To assess their inorganic fraction, untreated OMW and the different samples obtained by the application of membrane purification processes were analyzed for their conductivity. The conductivity of the different samples vs. the concentration level expressed as the volume concentration ratio is reported in Figure 3. The conductivity of untreated OMW was 7 mS/cm which corresponds to a concentration of total dissolved salt (TDS) of about 4 g/L expressed as NaCl equivalent. The sample collected after microfiltration (MF1 sample, Figure 2) showed a similar value, thus confirming that this purification step did not alter the salt content of the sample. RO selectively retains almost all solutes, allowing the exclusive passage of water. In our experiments the volume concentration factor was 7.3, and the conductivity of the concentrate (RO1 sample, Figure 2) increased to 30 mS/cm which corresponds to a TDS of about 19 g/L of NaCl equivalent. Vacuum membrane distillation (VMD) further concentrated the OMW reverse osmosis fraction achieving an overall volume concentration ratio of about 15 with a final conductivity of about 41.8 mS/cm (TDS = 27 g/L NaCl equivalent) for MD1 and MD2 fractions (Figure 2).

To assess the efficacy of RO and VMD processes to retain the dissolved inorganic electrolytes, two samples of permeate water were collected at the middle (ROp1 sample, Figure 2) and at the end (ROp2 sample, Figure 2) of the RO concentration process. Similarly, the initial and final distillate samples (MDd1 and MDd2, Figure 2) from the VMD concentration step were considered. The conductivity values of these samples (Table 1) confirmed that both methods reduced the electrolyte content in the fraction by about 98.5%.

**Figure 4:**
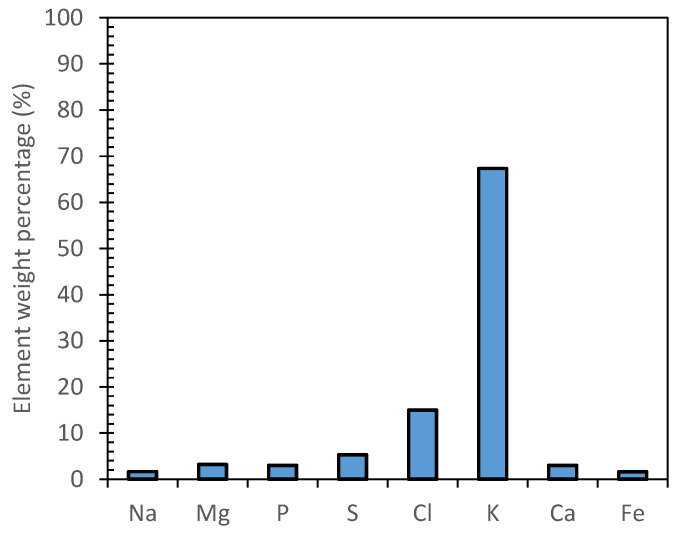
EDX semiquantitative analysis of representative sample.

The inorganic species composition of MF1, RO and MD samples was investigated through drying and calcination at 600 °C and semiquantitative analysis. All the analyzed samples showed the same inorganic average composition of the inorganic residue (Figure 4). A relevant mass concentration of potassium (ca 67%) was found, followed by chlorine (ca 15%). Other elements (i.e., Na, Mg, P, S, Ca and Fe) were observed in comparable amounts with a weight percentage below 7%. 

### 3.2. OMW Characterization

#### 3.2.1. FTIR Analysis

**Figure 5:**
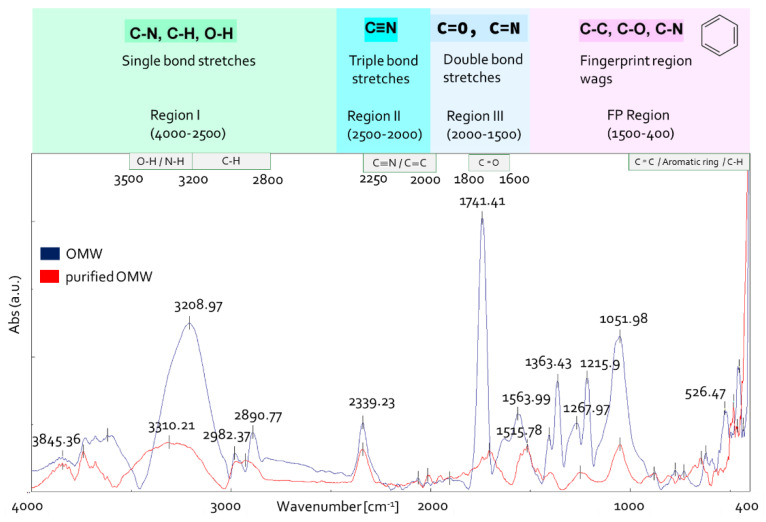
OMW IR spectra before and after purification.

FTIR spectroscopy is considered an effective and advantageous analytical method to study the functional groups of the organic compounds in OMW [39,40]. To assess whether the purification process affected OMW composition, untreated OMW and RO1 sample (Figure 2) were analyzed with IR spectroscopy (Figure 5). The IR spectra were analyzed according to the literature data [41,42,43,44].

The IR spectral examination of unprocessed OMW (Figure 5) revealed a large band in the range of 3500 to 3000 cm^−1^ which was attributed to OH hydroxyl group stretching vibrations (alcohols, phenols and carboxylic acids, 3670–2450 cm^−1^) [44,45]. The bands at 2980 and 2890 cm^−1^ are due to stretching vibrations of aliphatic C-H of CH_2_ and CH_3_ groups. The small absorption band at 2340 cm^−1^ is due to atmospheric CO_2_ present in the sample chamber during the collection of data. An intense band was found in spectral region III, centered at 1740 cm ^−1^. This band could correspond to valance vibrations C=O of carboxyl, ketone groups and esters [46]. In the fingerprint region 1500–400 cm^−1^, the bands at 1363 cm^−1^ are probably related to C–H bending of CH_3_ groups or to COO− antisymmetric stretching and vibration of C=O or deformation of C-H [47]. The signal at 1216 cm^−1^ indicates C-O stretching of aryl ethers and phenols and the stretching vibration of aromatic C_ar_-O and/or in-plane deformation of CO_2_H in carboxylic acids or unsaturated esters [48], and the band at 1052 cm^−1^ can be related to vibration in carbohydrates, aromatic ethers and polysaccharides [49]. 

The comparison of IR spectra between treated and untreated OMW highlighted that the intensity and the number of bands are reduced in purified OMW (Figure 5). A band can be observed at 1516 cm^−1^ in the spectrum of purified OMW, which is due to stretching vibration of C_ar_=C_ar_ in polar aromatic group type phenols and to flavonoids and aromatic rings (stretching of aromatic C=C) [39]. 

#### 3.2.2. TPs and DPPH Radical-Scavenging Activity in OMW

**Figure 6:**
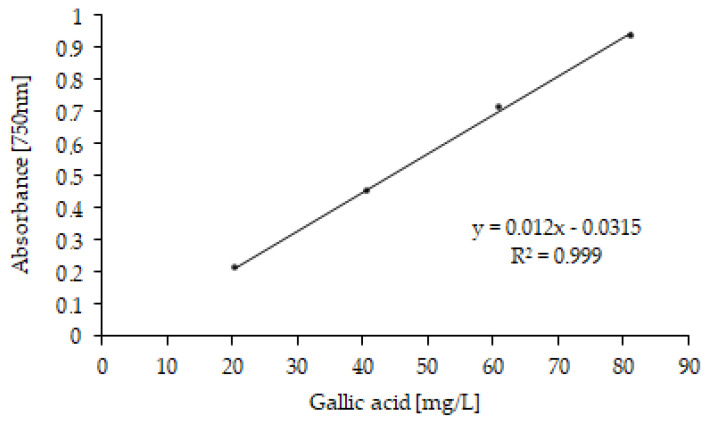
Gallic acid calibration curve (concentrations ranging from 20 to 80 mg/L).

The total polyphenol (TP) content, expressed as mg equivalent of gallic acid (GAE) per L of sample [50,51], was determined for MF, RO, ROp, MD and MDd fractions (Table 2). As OMW composition is affected by numerous factors such as the extraction method, the type and maturity of the olives, the region of origin and climatic conditions, the TP content of a microfiltration fraction obtained from different olive oil campaigns was determined (MF2 fraction). A linear gallic acid regression is shown in Figure 6.

**Figure 7:**
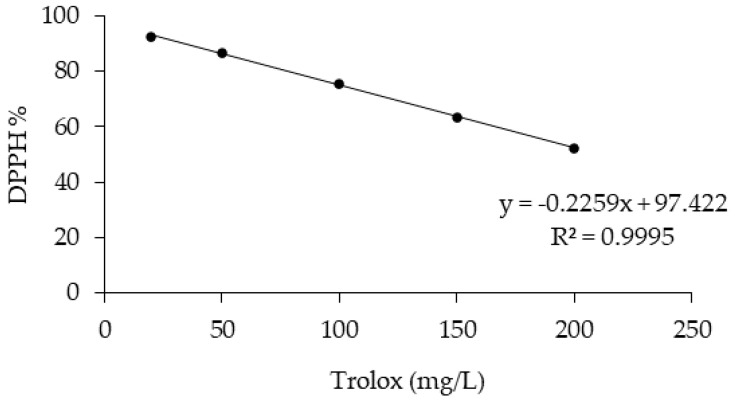
Trolox calibration curve (concentrations ranging from 20 to 200 mg/L).

The TP contents were found to be related to the concentration level of the fraction, and the MD2 sample displayed the highest quantity of polyphenols (15.38 g GAE/L, *p* < 0.001). A similar trend emerged for the antioxidant activity (AA%, *p* < 0.001, Table 3, Figure 7), and the two MD fractions, obtained from the final VMD purification step, showed the highest AA percentage values.

Interestingly, the microfiltration fractions collected in two different olive oil campaigns (i.e., MF1 and MF2) showed significant difference (*p* < 0.001) in their TP contents while still maintaining similar antioxidant activity.

### 3.3. Antibacterial Activity

The antibacterial activity of all OMW purified fractions (namely MF1, RO1, ROP1, ROP2, MD1, MD2, MDd1 and MDd2 samples, Figure 2) was preliminary evaluated against a panel of 12 bacterial strains, representative of the most clinically relevant Gram-positive and Gram-negative species. The considered pathogens include Gram-positive *S. aureus* (two strains, one methicillin-sensitive and one methicillin-resistant), *S. epidermidis* (two strains, one methicillin-sensitive and one methicillin-resistant) and Gram-negative *E. faecalis* (two strains, one vancomycin-sensitive and one vancomycin-resistant), *E. faecium* (two strains, one vancomycin-sensitive and one vancomycin-resistant isolate), *E. coli* (two strains, one was a New Delhi metallo-β-lactamase (NDM)-producing isolate) and two *P. aeruginosa* MDR isolates. Out of this preliminary screening, only the ROp1 fraction emerged as ineffective (MIC value > 128 mg/mL) against all the considered bacterial strains whereas MF1, RO1, ROP2, MD1, MD2, MDd1 and MDd2 samples showed significant antibacterial activity (MIC value range: 8–125 mg/mL, data not shown) on selected strains.

Therefore, the ROp1 fraction was not further investigated, and the seven active OMW samples were tested against 39 Gram-positive and Gram-negative isolates that included clinically relevant, multi-drug-resistant (MDR) strains. Additionally, a strain of the phytopathogen *Pseudomonas syringae* pv. *tomato* was included in the panel (Table 4 and Table 5). All analyzed fractions showed a widespread antibacterial activity against both antibiotic-susceptible and antibiotic-resistant Gram-positive and Gram-negative strains. This aspect is particularly relevant, given the considerable structural differences between Gram-negative and Gram-positive bacteria that account for distinct antibiotic susceptibility and resistance mechanisms [52,53,54]. The MD2 sample proved to be active against Gram-positive pathogens and showed the lowest MIC values (8–16 mg/mL) against Gram-negative bacteria including colistin-resistant *P. aeruginosa* 265, *M. morganii* 372, *P. stuarti* 374 and *S. marcescens* 400 strains, causative agents of difficult-to-treat, clinically relevant infections. All OMW samples were also active against *P. syringae* pv. *tomato*, an important seedborne pathogen known to causes bacterial speck disease in tomato [55]. The bacterium can also infect crucifers, and some strains are pathogens of the model plant *Arabidopsis thaliana*. Tomato bacterial speck is one of the most serious and feared plant diseases in the world and is characterized by the presence of fatty and dark spots, initially small, that quickly become brown to black in color on the leaflets, stems and fruit and reduce the quantity and quality of fruit yield [56]. Even against this phytopathogen, the MD2 sample was found to be the most active fraction with a MIC value of 8 mg/mL and an MBC value of 31 mg/mL.

Interestingly, the different antibacterial properties of the OMW purified fractions cannot be solely attributed to the different antioxidant activity of the samples. Thus, the highly active antibacterial MD2 sample (MIC range 8–16 mg/mL) was characterized by the highest AA% value (82.44%, Table 3), and MD1 (AA% = 46.9%) and RO1 fractions (AA% = 34.61%) showed similar anti-bacterial properties with MIC values in the 16–31 mg/mL range. However, despite the different AA% values, ROP2 (AA% = 26.9%) and MDd1 (AA% = 11.36%) showed similar MIC values in the 62–125 mg/mL range but proved to be less effective than the MDd2 fraction characterized by a reduced antioxidant activity (AA% = 10.74%). This observation is in accordance with published results that correlate the differences in antioxidant activities of OMV samples to the distinct phenolic profiles [39]. Similarly, the concentration of polyphenols cannot be clearly linked to the MIC values of the fractions. Thus, RO1 and MD1 samples contain a different quantity of polyphenols (TP values 8.9 and 6.5 g GAE/L, respectively) still showing similar MIC values (16–31 mg/mL). The MDd2 sample (TP = 0.01 gGAE/L, MIC range: 31–62 mg/mL) proved to be as effective as RO1 and MD1 against bacteria growth even though it contained a smaller quantity of total polyphenols. Furthermore, the MDd2 fraction was more active than the ROP2 sample (MIC 125–62 mg/mL), characterized by a higher polyphenol content (TP = 0.05 gGAE/L, Table 2). 

To verify the potential effect of KCl concentration on the antibacterial activity of OMW samples (Figure 3 and Figure 4), a 40 g/L (0.5 M) KCl solution was tested against all the bacteria strains. The MIC values obtained (data not shown) clarified that the growth of all considered Gram-positive and Gram-negative species was not inhibited by the inorganic residue, particularly K^+^, even at such a high concentration of KCl.

The favorable and powerful synergistic combination of the different components of the OMV samples was also evident from the minimum bactericidal concentration (MBC) values (Table 4 and Table 5). The MD2 sample emerged as the most active fractions with an excellent bactericidal capacity for Gram-positive and Gram-negative species.

Finally, the adopted testing approach represents an innovative strategy in the field of OMW. The available literature usually focuses on the antibacterial activity of phenolic extracts or phenolic enriched extracts obtained from OMW rather than on OMW as obtained from the purification procedure [57,58,59]. Additionally, the available microbiological investigations usually lack a quantitative determination of the MIC values and report only qualitative information. A rare quantitative study was reported by Roila et al. [60]. The authors evaluated the antibacterial activity of OMW polyphenol extracts against 65 strains of *P. fluorescens* (including also the ATCC 13525 strains) isolated from mozzarella cheese. The determined MIC values were in accordance with those assessed for the MD2 sample.

## 4. Conclusions

Through an innovative process that sequentially combines three membrane-based strategies (MF, RO and VMD), different OMW fractions were isolated and characterized. The eight analyzed fractions showed distinct antioxidant activities and various total polyphenol contents that correlate with a different qualitative composition, as highlighted by IR analyses. 

The antibacterial potency of the different OMW samples (considered as whole mixtures) was evaluated in a quantitative assay. With the sole exception of the ROp1 fraction, all analyzed samples showed significant antibacterial activity against clinically relevant Gram-positive and Gram-negative pathogens, with MD2 being the most active sample. Furthermore, the analyzed portions proved to be effective against the growth of *P. syringae* pv. *tomato*, an important seedborne pathogen known to causes bacterial speck disease in tomato. 

Overall, the collected data indicate OMW as a valuable byproduct of olive oil production process that can be valorized through a suitable purification process. The antibacterial effects of OMW extracts represent a promising area for therapeutic purposes in the human environment, particularly since the individual phenolic compounds appear to offer greater activity when administered as an extract than when used in purified form.

## Figures and Tables

**Table 1:** Conductivity of ROp and MDd samples.

Sample	Conductivity (mS/cm)
ROp1	0.14
ROp2	1.00
MDd1	0.42
MDd2	0.63

**Table 2:** Total phenolic content analyzed in OMW using the Folin–Ciocalteu assay (mean value ± standard deviation (SD) of three independent experiments (*n* = 3), A = absorbance values). Numbers followed by different letters are statistically different at *p* < 0.001 (Tukey’s test).

Sample Name	A (λ = 750 nm)	g GAE/L
MF1	0.188	1.304 ± 0.150 ^d^
MF2	0.333	0.251 ± 0.056 ^e^
ROP1	0.042	0.001 ± 0.001 ^e^
ROP2	0.097	0.055 ± 0.006 ^e^
RO1	0.131	8.292 ± 0.251 ^b^
MD1	0.110	6.542 ± 0.227 ^c^
MD2	0.216	15.375 ± 0.015 ^a^
MDd1	0.180	0.012 ± 0.001 ^e^
MDd2	0.150	0.010 ± 0.001 ^e^

**Table 3:** Evaluation of antioxidant activity percent (AA%) using DPPH assay (mean value ± standard deviation (SD) of three independent experiments (*n* = 3)). Numbers followed by different letters are statistically different at *p* < 0.001 (Tukey’s test).

Sample Name	A (517 nm)	DPPH%	AA%
MF1	0.861	88.95 ± 0.3	11.05 ± 0.3 ^e^
MF2	0.893	92.25 ± 0.2	7.75 ± 0.2 ^f^
ROP1	0.898	92.77 ± 0.4	7.23 ± 0.4 ^f^
ROP2	0.708	73.14 ± 0.1	26.86 ± 0.1 ^d^
RO1	0.633	65.39 ± 0.2	34.61 ± 0.2 ^c^
MD1	0.514	53.10 ± 0.2	46.90 ± 0.2 ^b^
MD2	0.17	17.56 ± 0.3	82.44 ± 0.3 ^a^
MDd1	0.858	88.64 ± 0.5	11.36 ± 0.5 ^e^
MDd2	0.864	89.26 ± 0.1	10.74 ± 0.1 ^e^

**Table 4:** MIC and MBC values expressed as mg/mL of the seven OMW samples on the selected Gram-positive strains. Experiments were carried out in triplicate. The degree of concordance in all the experiments was 3/3. Variation among triplicate samples was less than 10%. MRSA: methicillin-resistant *S. aureus* strains; MRSE: methicillin-resistant *S. epidermidis* strains VRE: vancomycin-resistant isolates.

	MF1		RO1		ROP2		MD1		MD2		MDd1		MDd2	
	MIC	MBC	MIC	MBC	MIC	MBC	MIC	MBC	MIC	MBC	MIC	MBC	MIC	MBC
*S. aureus*														
17 MRSA	125	>125	16	31	125	>125	16	31	8	16	125	>125	62	>125
18 MRSA	125	>125	16	31	125	>125	16	16	8	16	125	>125	62	>125
187 MRSA	125	>125	31	31	125	>125	16	31	8	16	125	>125	62	>125
188 MRSA	125	>125	16	31	125	>125	16	16	8	16	125	>125	62	>125
*S. epidermidis*														
22 MRSE	125	>125	16	31	125	>125	16	16	8	16	125	>125	62	125
180 MRSE	62	>125	16	16	125	>125	16	16	8	16	62	125	62	62
181 MRSE	62	>125	16	16	125	>125	16	16	8	8	125	>125	62	125
222 MRSE	125	>125	16	16	125	>125	16	16	16	16	125	>125	62	125
*E. faecalis*														
1 VRE	125	>125	16	62	125	>125	16	62	16	31	62	>125	62	>125
4	125	>125	16	62	125	>125	16	62	16	31	62	>125	31	>125
50 VRE	125	>125	16	62	125	>125	16	62	16	31	62	>125	62	>125
365 VRE	125	>125	16	125	125	>125	16	125	16	31	62	>125	31	>125
*E. faecium*														
21	125	>125	16	62	125	>125	16	62	16	16	62	>125	62	>125
40	125	>125	16	62	125	>125	16	62	16	16	62	>125	62	>125
300 VRE	125	>125	16	62	125	>125	16	62	16	16	62	>125	62	>125
362 VRE	125	>125	16	62	125	>125	16	62	16	16	62	>125	31	>125

**Table 5:** MIC and MBC values expressed as mg/mL of the seven OMW samples on the selected Gram-negative strains. Experiments were carried out in triplicate. The degree of concordance in all the experiments was 3/3. Variation among triplicate samples was less than 10%. C denotes resistance to colistin; * denotes a class A carbapenemase (KPC)-producing bacterium.

	MF1		RO1		ROP2		MD1		MD2		MDd1		MDd2	
	MIC	MBC	MIC	MBC	MIC	MBC	MIC	MBC	MIC	MBC	MIC	MBC	MIC	MBC
*P. aeruginosa*														
403	62	>125	16	31	62	>125	16	31	8	16	62	>125	31	125
432	62	>125	16	31	62	>125	16	31	8	16	62	>125	31	125
265c	125	>125	16	31	125	>125	16	31	16	16	62	>125	31	125
1	125	>125	16	31	125	>125	16	31	16	16	62	>125	31	125
2v	62	>125	16	31	62	>125	16	31	8	16	62	>125	31	125
19v	125	>125	16	31	125	>125	16	31	8	16	62	>125	31	125
16b	125	>125	16	31	125	>125	16	31	16	16	62	>125	31	125
12b	62	>125	16	31	125	>125	16	31	8	16	62	>125	31	125
8g	125	>125	16	31	62	>125	16	31	8	16	62	>125	31	125
*A. baumannii*														
245	125	>125	16	31	125	>125	16	31	16	16	62	125	31	125
*M. morganii*														
372	125	>125	16	31	125	>125	16	31	16	31	62	>125	31	125
*P. stuarti*														
374	125	>125	16	31	125	>125	16	31	16	16	62	>125	62	125
*K. pneumoniae*														
375 *	125	>125	31	62	125	>125	31	62	16	31	62	>125	62	125
376 *	62	>125	16	62	62	>125	16	62	8	31	62	>125	62	>125
377 *	125	>125	31	31	125	>125	31	62	16	31	62	>125	62	>125
*S. marcescens*														
400	125	>125	31	31	125	>125	16	31	16	31	62	>125	31	125
*S. maltophilia*														
391	62	>125	16	31	62	>125	16	31	8	16	62	>125	31	125
392	62	>125	16	31	62	>125	16	31	16	16	62	>125	31	125
*E. coli*														
224	125	>125	31	62	125	>125	31	62	16	31	62	>125	62	>125
238 *	125	>125	31	62	125	>125	31	62	16	31	62	>125	62	>125
4	125	>125	31	62	125	>125	31	62	16	31	62	>125	62	>125
*P. syringae*														
266	62	>125	16	31	62	>125	16	31	8	31	62	>125	31	125

## Data Availability

The data presented in this study are available in the article.
